# Adaptive spatial-channel feature fusion and self-calibrated convolution for early maize seedlings counting in UAV images

**DOI:** 10.3389/fpls.2024.1496801

**Published:** 2025-02-03

**Authors:** Zhenyuan Sun, Zhi Yang, Yimin Ding, Boyan Sun, Saiju Li, Zhen Guo, Lei Zhu

**Affiliations:** ^1^ School of Civil and Hydraulic Engineering, Ningxia University, Yinchuan, China; ^2^ Key Laboratory of Digital Water Governance for Yellow River Water Network, Yinchuan, China; ^3^ Field Scientific Observation and Research Station of Agricultural Irrigation in Ningxia Diversion Yellow Irrigation District, Ministry of Water Resources, Yinchuan, China; ^4^ The Scientific Research Institute of the Water Conservancy of Ningxia, Yinchuan, China

**Keywords:** early maize seedlings counting, adaptive spatial-channel feature fusion, self-calibrated convolution, RC-Dino, deep learning

## Abstract

Accurate counting of crop plants is essential for agricultural science, particularly for yield forecasting, field management, and experimental studies. Traditional methods are labor-intensive and prone to errors. Unmanned Aerial Vehicle (UAV) technology offers a promising alternative; however, varying UAV altitudes can impact image quality, leading to blurred features and reduced accuracy in early maize seedling counts. To address these challenges, we developed RC-Dino, a deep learning methodology based on DINO, specifically designed to enhance the precision of seedling counts from UAV-acquired images. RC-Dino introduces two innovative components: a novel self-calibrating convolutional layer named RSCconv and an adaptive spatial feature fusion module called ASCFF. The RSCconv layer improves the representation of early maize seedlings compared to non-seedling elements within feature maps by calibrating spatial domain features. The ASCFF module enhances the discriminability of early maize seedlings by adaptively fusing feature maps extracted from different layers of the backbone network. Additionally, transfer learning was employed to integrate pre-trained weights with RSCconv, facilitating faster convergence and improved accuracy. The efficacy of our approach was validated using the Early Maize Seedlings Dataset (EMSD), comprising 1,233 annotated images of early maize seedlings, totaling 83,404 individual annotations. Testing on this dataset demonstrated that RC-Dino outperformed existing models, including DINO, Faster R-CNN, RetinaNet, YOLOX, and Deformable DETR. Specifically, RC-Dino achieved improvements of 16.29% in Average Precision (AP) and 8.19% in Recall compared to the DINO model. Our method also exhibited superior coefficient of determination (R²) values across different datasets for seedling counting. By integrating RSCconv and ASCFF into other detection frameworks such as Faster R-CNN, RetinaNet, and Deformable DETR, we observed enhanced detection and counting accuracy, further validating the effectiveness of our proposed method. These advancements make RC-Dino particularly suitable for accurate early maize seedling counting in the field. The source code for RSCconv and ASCFF is publicly available at https://github.com/collapser-AI/RC-Dino, promoting further research and practical applications.

## 1 Introduction

The precise and prompt quantification of early maize seedlings is of paramount importance to a plethora of agricultural operations, including yield forecasting, field management, and experimental research. As agricultural practices become increasingly sophisticated, the need for rapid, efficient, and precise acquisition of seedlings information has become paramount. Historically, the assessment of seedlings counts has been predominantly conducted through manual observation, a method that is inherently time-consuming, prone to human error, and increasingly unsuitable for meeting the demands of contemporary large-scale agricultural systems (Liu et al., 2016; Weiss et al., 2016a; Madec et al., 2019; Maheswari et al., 2021).

In recent years, the development of computer vision, machine learning, deep learning, and other technologies has provided novel approaches to addressing the challenge of seedling counting in agriculture. In the field of computer vision, traditional image processing techniques, including superpixel generation, threshold segmentation, and morphological operations, have been extensively employed in plant phenotyping (Xiong et al., 2017). Despite the automation of the seedling counting process to a certain extent, these methods remain susceptible to complex backgrounds and variations in lighting conditions. The application of machine learning techniques, in particular random forests and support vector machines, has led to notable improvements in the accuracy and efficiency of seedling counting. This is achieved by extracting features from images and constructing predictive models (Peng et al., 2023). However, these methods typically necessitate the provision of a substantial amount of manually labelled data and can be computationally expensive when dealing with high-resolution images. Deep learning methods, such as Convolutional Neural Networks (CNNs) and Mask R-CNN, employ an end-to-end learning framework that enables the automatic extraction of intricate features, thereby achieving high-precision seedling detection and counting (Hasan et al., 2018; Peng et al., 2023). These methods demonstrate remarkable efficacy when processing large-scale datasets, yet they require substantial computational resources. Moreover, alternative methodologies, such as video-based tracking techniques (Tan et al., 2023) and ensemble learning methods (Zhao et al., 2023), have exhibited distinctive capabilities in seedling counting, effectively addressing the challenges posed by dynamic environments and complex backgrounds. The advancements in these technologies provide diverse options for achieving rapid and accurate seedling counting, thereby advancing the progress of modern agricultural technology.

Two principal categories of object detection algorithms are distinguished: two-stage detectors, exemplified by Faster R-CNN (Ren et al., 2017), and single-stage detectors, represented by methods such as YOLOX (Zheng et al., 2021) and RetinaNet (Lin et al., 2020). In detection-based approaches, the counting outcomes are typically derived through the aggregation of detected bounding boxes across images (Zhang and Li, 2023), or by enumerating foreground objects within video sequences (Shen et al., 2023). These methodologies often necessitate manual parameter tuning for components such as non-maximum suppression (NMS) and anchor design, which can significantly influence their accuracy.

In 2020, Carion et al. (2020) introduced DETR, a transformer-based model that eliminates the necessity for manual adjustment of components such as NMS and anchors. This approach achieved competitive performance with Faster R-CNN on the COCO dataset. However, it should be noted that DETR displays a certain degree of limitation in terms of its capacity to detect smaller objects, in comparison to Faster R-CNN. To address this shortcoming, Zhang et al. (2022) proposed the Dino model, an enhancement of DETR. This model incorporates several innovations, including a multi-head attention mechanism, a multi-scale fusion module, and contrastive denoising training (CDN), which collectively address challenges associated with multi-scale feature integration and optimization efficiency. The Dino model demonstrates improved counting performance over its predecessor.

Despite the significant advancements in computer vision techniques for handling larger objects, there remain notable limitations when applying these technologies to specific domains such as corn production. Existing methods struggle particularly with the detection and counting of small targets like early maize seedlings in large-scale agricultural images captured by drones flying at a higher flight altitude. As the flight altitude increases, the image features of early maize seedlings become more small, imposing higher demands on object detection algorithms. These small features are prone to being overlooked during the feature extraction process, leading to a notable decrease in counting accuracy. Consequently, this limitation restricts the potential application of drone technology in modern agriculture.

To address the aforementioned challenges, this paper proposes an improved version of the Dino model, termed RC-Dino, aimed at enhancing the accuracy and efficiency of early maize seedlings detection. The primary innovation of our model lies in the adoption of RSCconv convolution, a spatial self-calibration convolution technique inspired by Liu et al. (2020), which enables more precise calibration and localization of features in the spatial domain. This capability effectively distinguishes early maize seedlings from the background and other non-target objects. Furthermore, to optimize feature representation across different scales, we integrate an Advanced Spatial Contextual Feature Fusion (ASCFF) module into the Neck section of the model. This module is an enhanced version of the Adaptive Spatial Feature Fusion (ASFF) proposed by Liu et al. (2019), designed to better integrate multi-scale features extracted from the backbone network, thereby strengthening the feature representation of early maize seedlings. Lastly, by employing transfer learning strategies and leveraging pre-trained backbone network weights, we not only accelerate the training process but also improve the generalization ability and ultimate detection accuracy of the model.

To evaluate the efficacy and seamless integration of this model, we conducted an assessment of its performance in detection and counting using RSCconv, ASCFF, and the transfer learning approach. Subsequently, in the same task, we conducted a comparative analysis of our model’s performance with several classic models. Then, we introduced RSCconv and ASCFF into object detection models such as Faster R-CNN, RetinaNet and Deformable DETR and conducted a comparative analysis. In consideration of the environmental conditions that prevail in the northern provinces of China, where maize is extensively cultivated in arid and semi-arid regions with low annual rainfall and fewer instances of cloud formation, resulting in a higher proportion of sunny days, this study concentrated exclusively on the model’s counting performance under sunny conditions with high light intensity.

## 2 Materials and methods

### 2.1 Data acquisition

The experimental data were collected at the Ningxia Center for Irrigation Experiments located in Xixia District, Yinchuan City, Ningxia Hui Autonomous Region, China. This center is located in the arid inland region of Ningxia, characterized by a typical temperate continental climate with an annual average precipitation of 195 mm and an annual sunshine duration ranging from 2,800 to 3,000 hours. The experimental plot features sandy soil.

**Figure 1:**
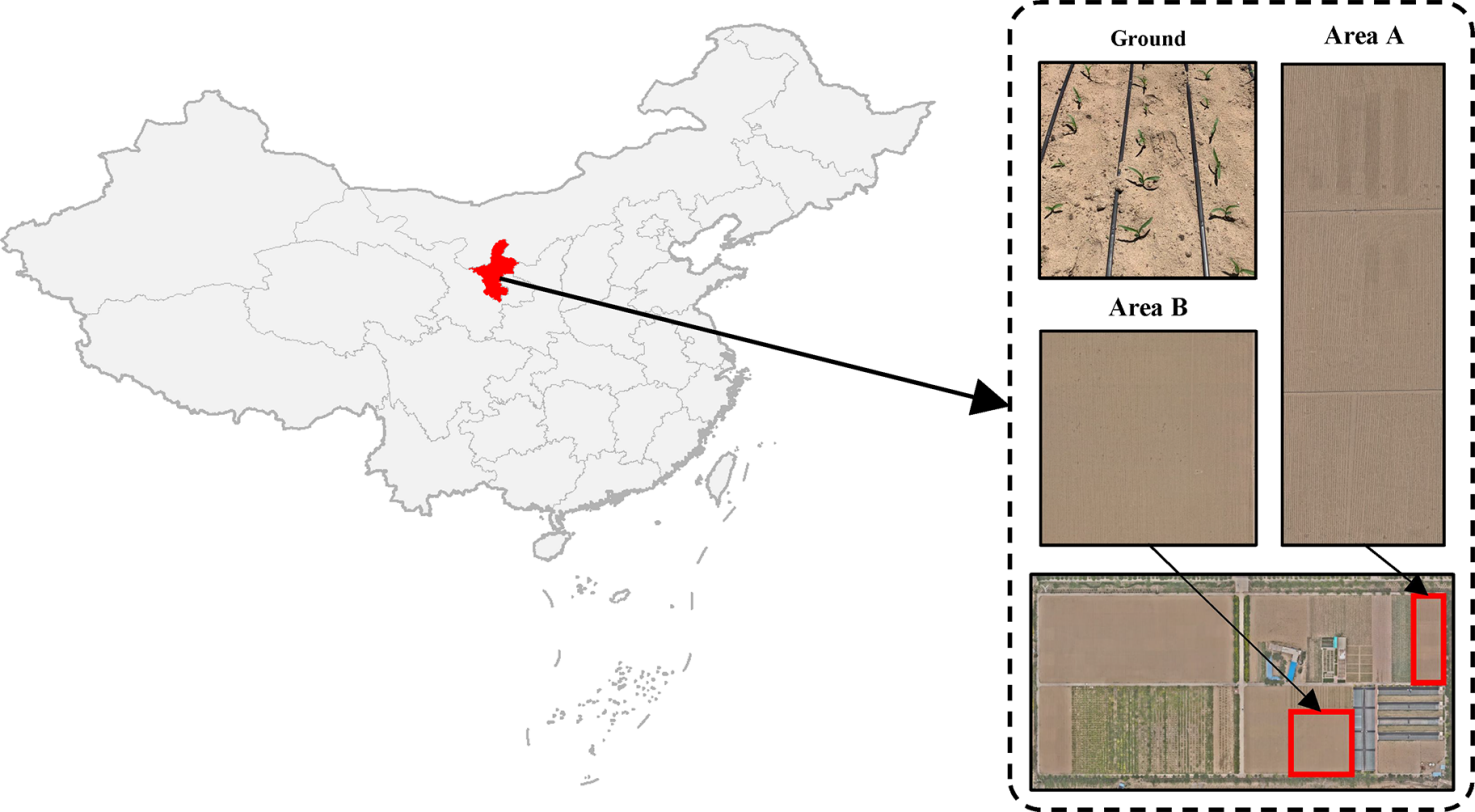
General situation of maize and experimental field. **(A)** The star represents the growing site of maize of accessions (Xixia District, Yinchuan City, Ningxia Hui Autonomous Region, China). **(B)** Close-up and aerial images of field experiments.

The experimental site comprises two areas, with area A measuring 3,776 m^2^ and area B measuring 5,016 m^2^. Data collection was conducted using DJI Mavic 3M UAV on May 25, 2023, and June 1, 2024, between the hours of 11:30 AM and 2:00 PM for both sub-plots. **Figure 1** provides an illustration of the distribution of these sub-plots.

In order to minimize the influence of light intensity on the detection effect, data was gathered in conditions of clear skies and minimal wind, at a time when all the maize plants had reached the seedlings stage. The UAV were equipped with a 20-megapixel camera and were set to stationary hover capture, with a flight height of 12 m (Area A), 20 m (Area B) and 24 m (Area A), an overlap rate of 80% for the side and heading, a pixel resolution of 0.32 cm (Area A), 0.54 cm (Area B) and 0.64 cm (Area A), and an image resolution of 5,280 × 3,956 pixels. A total of 876 images of early maize seedlings were obtained. For illustrative purposes, an example of early maize seedlings images is provided in **Figure 1**.

### 2.2 Data preprocessing and augmentation

#### 2.2.1 Data preprocessing

**Figure 2:**
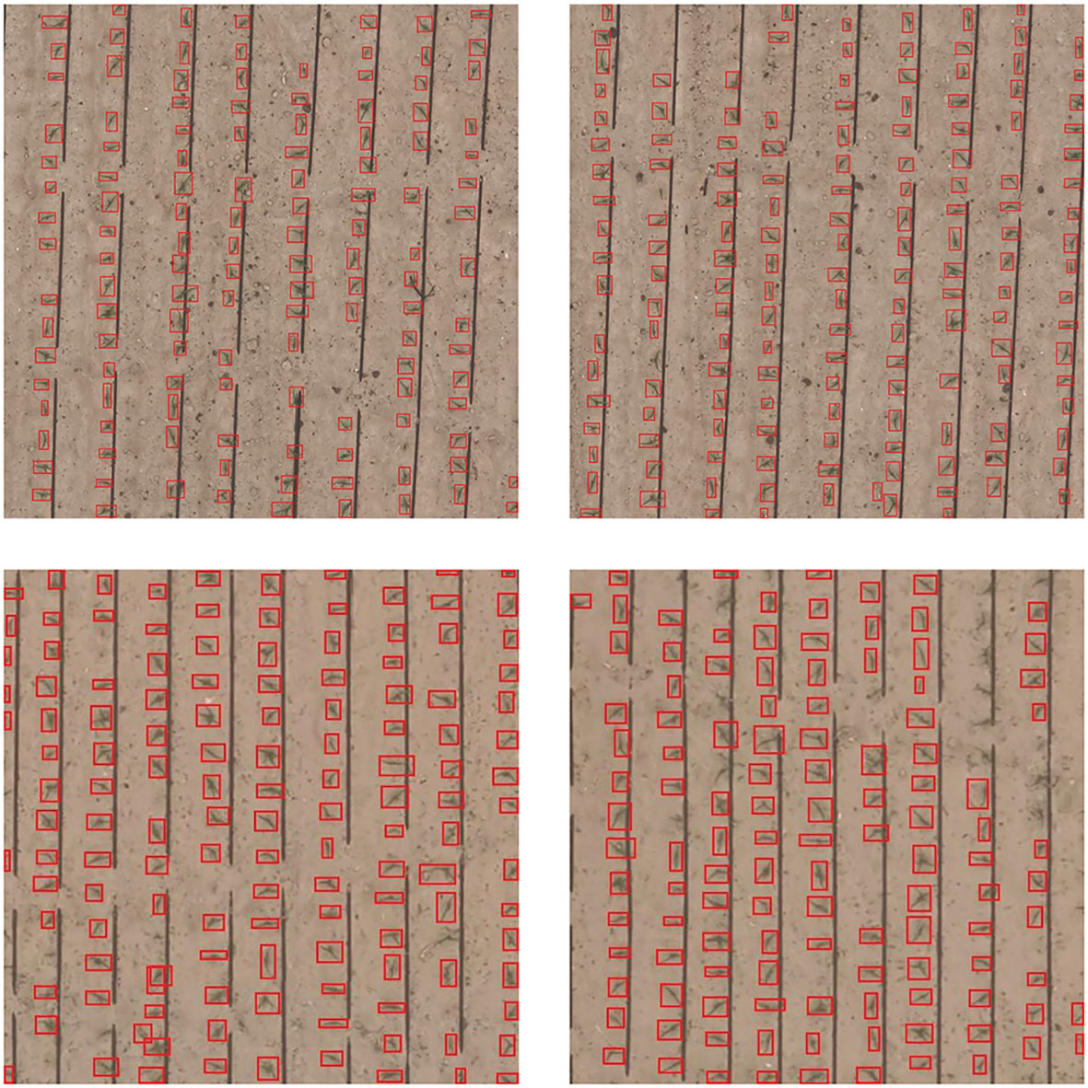
Illustrative image of dataset with featuring annotated information (512×512).

The UAV images of the area were processed using Pix4Dmapper version 4.4.9 (Pix4D SA, Lausanne, Switzerland) image stitching software. However, due to the considerable dimensions of the images and the limited extent of the early maize seedlings, direct training and detection proved to be impractical. Following the removal of irrelevant areas, the images of the research area were automatically cropped to a size of 512 × 512 pixels. A total of 1,233 early maize seedlings images were obtained. All early maize seedlings images were randomly divided into three sets: the training set, the validation set and the test set, in a ratio of 7:2:1. In order to assess the model’s ability to count at varying heights, 24-meter-high early maize UAV images were incorporated exclusively into the test set. Representative images from the Early Maize Seedling Detection (EMSD) dataset are presented in **Figure 2**.

The manual annotation of early maize seedlings was conducted using Labelme software (Russell et al., 2008) resulting in a total of 83,404 annotated seedlings. In accordance with the classification criteria for large, medium-sized, and small objects proposed by Lin et al. (2014), objects with an area of less than or equal to 32×32 pixels are classified as small, while those with an area greater than 32×32 pixels are classified as medium-sized. Pixels with an area of less than or equal to 96×96 are classified as medium-sized, while those larger than 96×96 are categorized as large. The distribution of small, medium and large objects in EMSD are shown in **Table 1**:

**Table 1:** Number and proportion of large, medium and small objects in EMSD.

Datasets	Small Objects		Medium-sized Objects		Large Objects	
	numbers	per	numbers	per	numbers	per
Train	44433	88.92%	5534	11.08%	0	0%
Val	12851	88.82%	1617	11.18%	0	0%
Test	10358	93.42%	730	6.58%	0	0%
Total	75523	89.56%	7881	10.44%	0	0%

#### 2.2.2 Data augmentation

In order to address the issue of model overfitting, which arises from the limited volume of data, an augmentation of the training set images and corresponding marking files is conducted utilizing techniques of data augmentation. This approach initially conducts random flipping operations, horizontally or vertically flipping the image with a 50% probability to facilitate the model’s adaptation to objects in various orientations. Subsequently, it adjusts the image size through a sequence of scale transformation operations, encompassing randomly selecting a scale from a predefined list for resizing the image, randomly cropping an area of the image, and once again randomly selecting a scale for resizing the image to a fixed size. These operations not only simulate diverse scenes and distances but also aid in enabling the model to capture more varied local features. Upon completion of these procedures, all data is amalgamated and incorporated into the dataset as the ultimate training dataset.

#### 2.2.3 Experimental configuration

The experiments in this study were conducted using an Ubuntu 18.04 LTS 64-bit operating system. The hardware setup consisted of four NVIDIA GeForce 3090 RTX graphics card and 128GB of memory. The CUDA version employed was 11.1, and the CUDNN version was 8.3.2. The object detection model was trained using the Python programming language and the PyTorch deep learning framework. For the training process, an initial learning rate of 0.002 was set. The AdamW optimizer was utilized to optimize the loss arising during the training process. The model was trained for 12 epochs with a batch size of 8. A weight decay of 0.0001 was applied. The learning rate for the Backbone was set to 0.1 times the initial learning rate.

#### 2.2.4 Counting performance evaluation methods for test dataset

**Figure 3:**
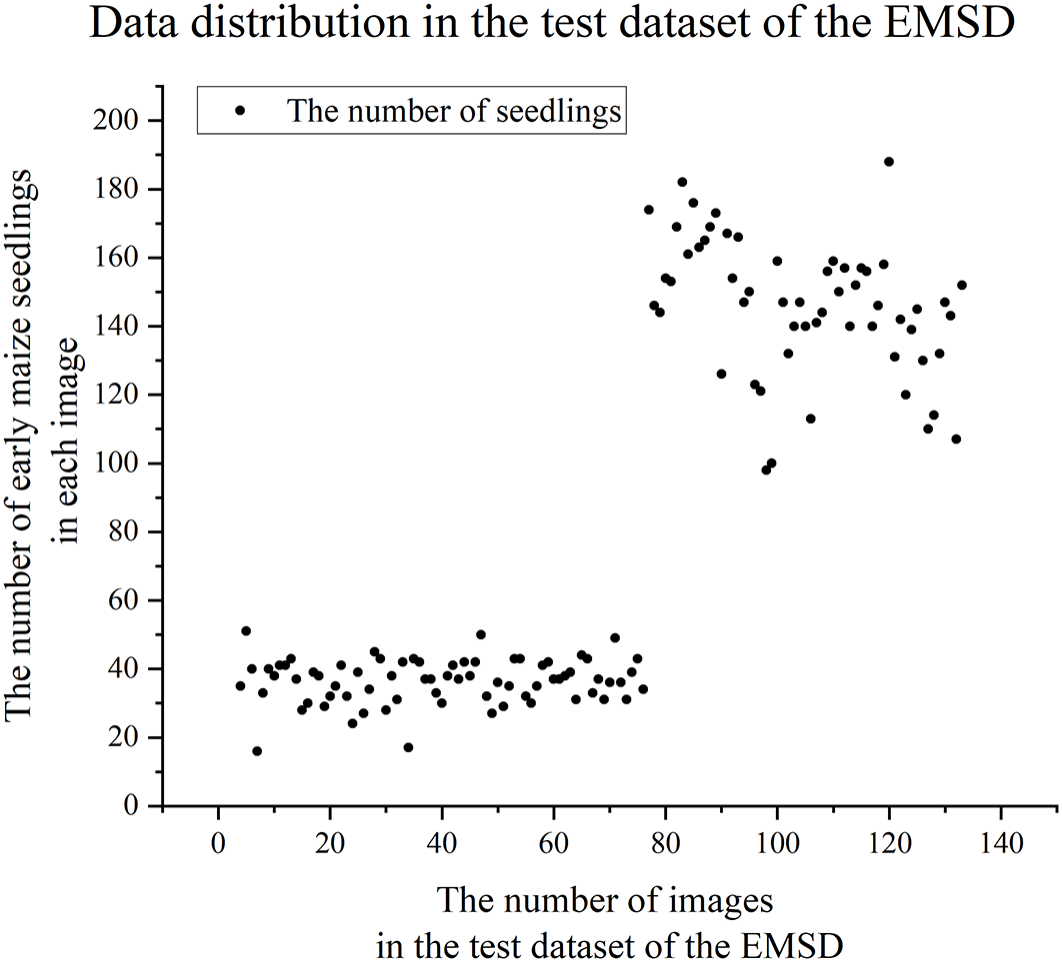
Data distribution in the test dataset of the EMSD.

In the EMSD dataset, compared to the training dataset and validation dataset, the test dataset contains drone images taken at higher flight altitudes. Through the analysis of the test dataset (see **Figure 3**), we observed that the number of early maize seedlings in the images had two significant distribution characteristics: one type of images had less than 80 seedlings, while the other type had more than 80 seedlings. In order to more accurately evaluate the model’s early maize seedlings counting performance at different flight altitudes, we divide the test dataset into two subsets: Test Dataset A (images with 80 or less seedlings, total 2773 seedlings) and Test Dataset B (images with more than 80 seedlings, total 8315 seedlings).

In the counting task, for Test Dataset A, a method based on mosaic of large image blocks was used to improve the accuracy of counting, and a 50% overlap rate was set to ensure that early maize seedlings in the edge region were not missed. For Test Dataset B, due to the low target density in the image, the model is used directly for inference without additional overlapping areas, thus simplifying the processing and improving computational efficiency.

### 2.3 RC-Dino structure and implementation details

Given the characteristics of the datasets in this study, such as high proportions of small and medium-sized objects, low-resolution early maize seedlings images, and limited early maize seedlings features, Dino, a representative algorithm in the DETR model family, is selected as the baseline model.

**Figure 4:**
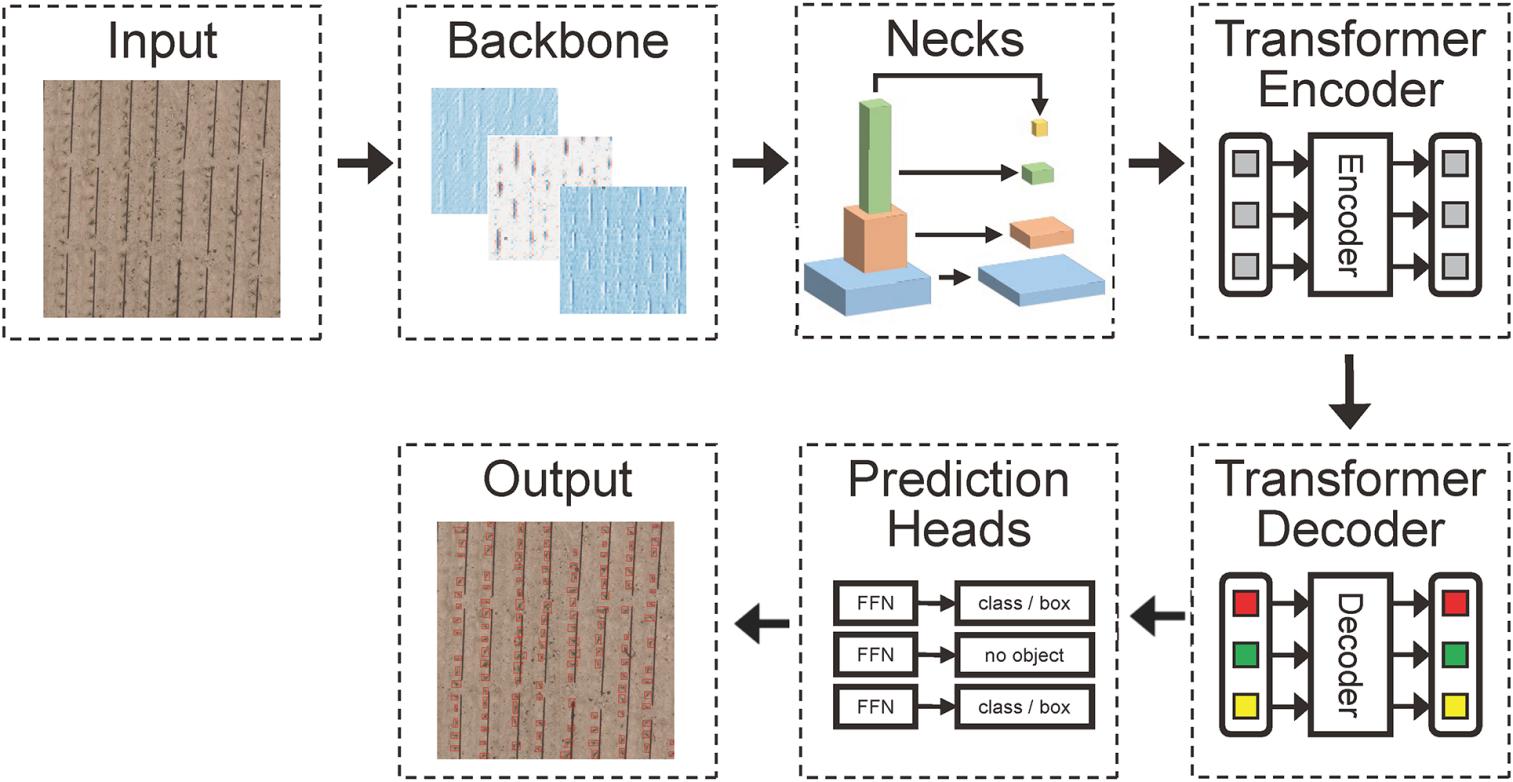
The architecture of the proposed of Dino Model.

The Dino model encompasses five crucial components: Backbone, Neck, Encoder, Decoder, and Prediction Heads. The Backbone harnesses the Resnet50 model (He et al., 2016), generate feature maps from Level 2, 3, and 4. Subsequently, the Neck together feature maps of disparate scales originating from the Backbone, passing them along to the Encoder. The role of the Encoder is to refine and streamline the feature maps obtained from the Neck. Following this, the Decoder amalgamates the processed feature maps with their corresponding real tags for decoding. Ultimately, the Prediction Heads output the conclusive results of the target detection. For illustration purposes, see **Figure 4**.

This section illuminates the construction and application of RSCconv and ASCFF, in addition to the employment of transfer learning techniques via pre-trained weights integrated into the Backbone, all aimed at enhancing the model’s detection and counting efficacy.

#### 2.3.1 RSCconv

To improve feature extraction for small objects such as early maize seedlings, we analyzed the ResNet50 backbone in the Dino model and found that its feature extraction for each relies mainly on 3×3 convolutions. Inspired by SCConv proposed by Liu et al. (2020), we designed RSCconv, a new convolutional structure that effectively detects and enhances the features of small objects. RSCconv’s internal communication mechanism enhances the early features of early maize seedlings, thus improving feature extraction for small objects without adding complexity.

**Figure 5:**
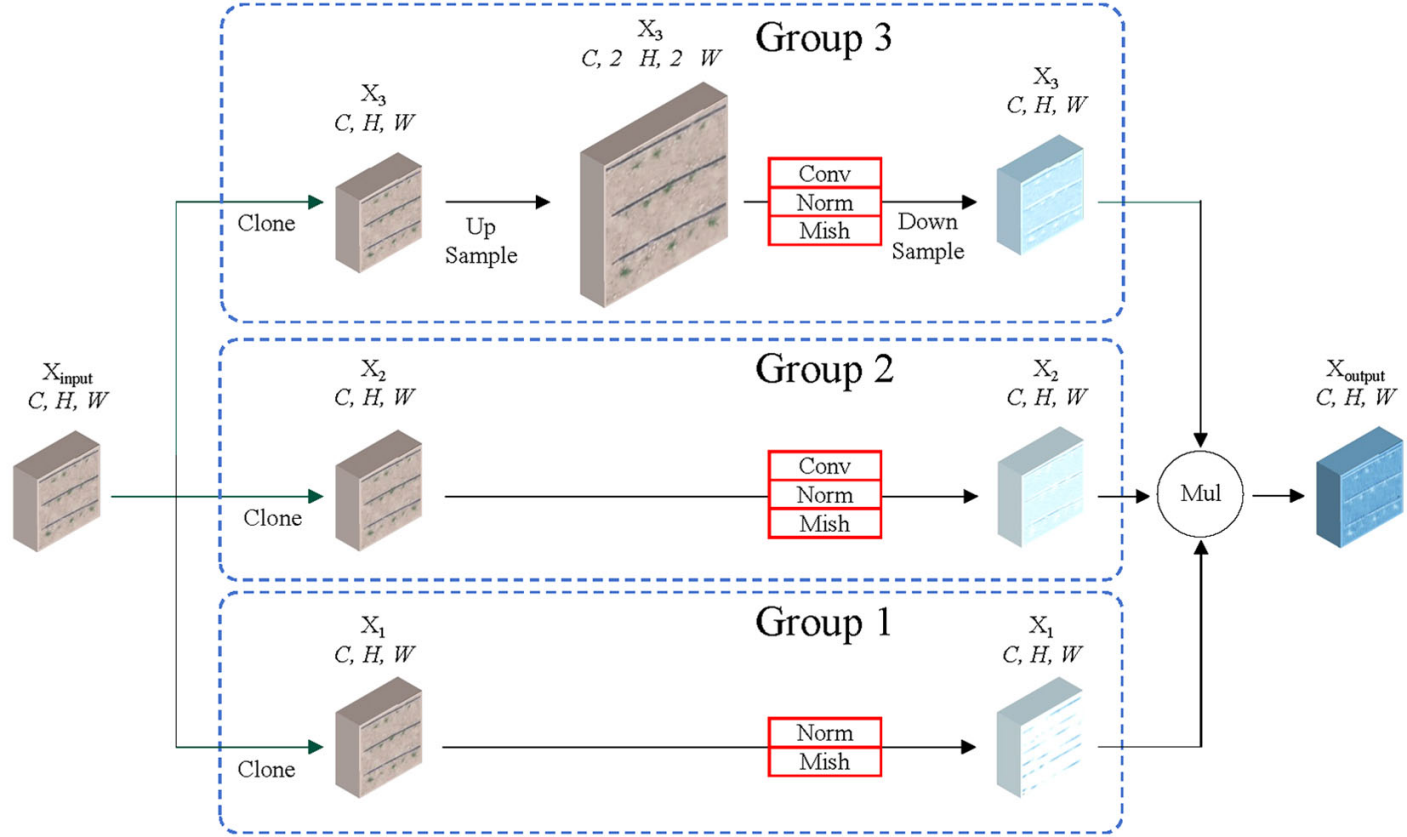
Structure diagram of RSCconv.

The design process is illustrated in **Figure 5**. The feature map is initially duplicated into three groups, with each group being inputted into a distinct path in order to collect different types of contextual information. The first group receives no treatment and is directly multiplied with the second and third groups. In this group, the residual connection concept from the ResNet model is employed to preserve the original information present in the feature maps, thereby preventing the loss of small object information that would otherwise occur during the feature extraction process. The second group employs a convolutional neural network with a 3*3 kernel for feature extraction and refinement of the early maize seedlings feature map. This enhances the features of small objects that have been extracted. The third group employs bilinear interpolation to upscale the early maize seedlings feature map, thereby increasing the spatial dimensions of the feature map and doubling the length and width in comparison to the original scale space. Following up sampling, the Conv(3x3) method is employed to refine the early maize seedlings feature map. This is then down sampled in order to map the feature map back to the original feature space. Finally, the feature maps from the first, second and third groups are fused through multiplication, thereby calibrating and highlighting the features of early maize seedlings after different processing steps.

The calibration operation in RSCconv enables each spatial position to adaptively incorporate its surrounding contextual information, thereby enhancing the expression of early maize seedlings features from the original scale space. The self-calibration function effectively enhances the representation of small objects, avoids contamination from irrelevant regions, and introduces different feature map information, which collectively serve to enhance the features of early maize seedlings.

#### 2.3.2 ASCFF

In the DINO model, the neck employs the ChannelMapper module. The module in question retrieves feature maps from levels 2, 3, and 4 of the backbone network, which are referred to as Level-1, Level-2, and Level-3 feature maps, respectively. Subsequently, each feature map is subjected to a convolutional process for the purpose of extracting features, after which a reduction in the number of channels is conducted, resulting in a total of 256 channels. Furthermore, the Level-3 feature map is convolved, down sampled, and channel-reduced to generate an output feature map with 256 channels and a size that is half that of the Level-3 feature map. However, due to the larger area of non-early maize seedlings in EMSD images compared to early ones, the feature extraction process frequently fails to identify small object features, which has a detrimental impact on the model’s ability to detect and count early maize seedlings.

**Figure 6:**
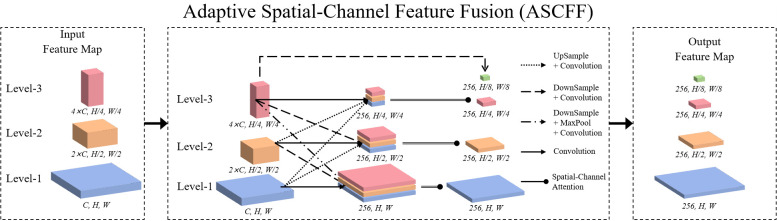
Structure diagram of ASCFF.

To address this issue, the model introduces an improved Adaptive Spatial-Channel Feature Fusion (ASCFF) mechanism, the details of the ASCFF module are shown in **Figure 6**. The ASCFF mechanism replaces the simple convolution operations performed by the ChannelMapper on all level feature maps. Initially, the Level-1, Level-2, and Level-3 feature maps are aligned to a uniform resolution. Subsequently, the model incorporates a spatial-channel attention mechanism that replaces the original adaptive algorithm for different feature maps, thereby enabling the adaptive adjustment of the relative importance of different feature maps. This facilitates a greater focus on more significant features, whereby the most informative features are highlighted while reducing noise, thereby enhancing target detection capabilities. This process not only strengthens early maize seedlings features but also suppresses non-maize seedlings features, thus enhancing the representation of early maize seedlings. The detailed steps are as follows:

Step 1: Harmonization of feature maps
  The feature maps obtained from the backbone at Levels 1, 2, and 3 are initially harmonized through up-sampling or down-sampling, ensuring that they possess identical spatial dimensions. Subsequently, the number of channels is standardized across these levels, facilitating subsequent processing.
Step 2: Fusion of aligned feature maps
  Following the harmonization process, the aligned feature maps from Levels 1, 2, and 3, which now possess consistent spatial dimensions and channel numbers, are concatenated. This concatenated feature map then undergoes a spatial-channel attention mechanism, which achieves feature fusion by emphasizing informative regions and channels while suppressing less relevant ones.
Step 3: Final feature processing
  The fused feature map, augmented with attention-enhanced data, is subjected to further processing through a feature extraction layer, resulting in the generation of the final output. This output encapsulates the salient information across multiple scales.

These operations permit the neck to retain high-resolution features from the superficial layers and high-semantic information from the deeper layers, enhancing key features while suppressing irrelevant ones, thereby enabling adaptive fusion. This results in an enhanced feature representation of the early maize seedlings, which effectively enhances the model’s detection accuracy.

#### 2.3.3 Transfer learning

In this study, we employed transfer learning, originally introduced by Weiss et al. (2016b), whereby pre-trained ResNet50 weights from ImageNet were transferred to the RC-Dino Backbone. By leveraging pre-trained backbone weights, we were able to reduce the time, data, and computational resources required for training, while also mitigating the overfitting issues that are commonly observed in small datasets and deep neural networks.

The 3x3 convolutions within each residual block of the backbone were replaced with RSCconv, which was designed to fit seamlessly into the original architecture and inherit the pre-trained weights. The backbone weights were kept unfrozen and initialized with pre-trained weights (where 3x3 convolution weights were inherited by RSCconv), which resulted in accelerated convergence and enhanced detection performance.

### 2.4 Evaluation metrics

In this study, a series of evaluation metrics were employed to assess the performance of deep learning algorithms. These included recall at different Intersection over Union (IoU) thresholds, average precision (AP) from 50% to 95%, and specific average precisions for small objects (AP_S_, with an area less than 32^2^) and medium-sized objects (AP_M_, with an area between 32^2^ and 96^2^). The aforementioned evaluation metrics were derived from the Dino model, as proposed by Zhang et al. (2022).

In the context of statistical analysis, the term ‘recall’ is defined as the probability of correctly predicting positive samples among all actual positive samples. This serves as an overall measure of prediction accuracy. The calculation is as follows:


(1) $$recall=\frac{TP}{TP+FN}$$

In this context, true positives (TP) represent the accurate identification of early maize seedlings, while false positives (FP) encompass instances where background or other features are erroneously classified as early maize seedlings. Conversely, false negatives (FN) refer to cases where early maize seedlings targets are incorrectly classified as other features.

The average precision (AP) is the mean value across all categories. In the case of the EMSD dataset, which contains only one category, AP represents the precision for that single category. AP is defined as the probability of actual positives among all samples predicted as positives. It measures the accuracy of positive sample predictions. The calculation is as follows:


(2) $$AP=\int_{0}^{1}P\left(R\right)dR$$

Where precision (P) computed as follows:


(3) $$precision=\frac{TP}{TP+FP}$$

The average precision (AP) at intersection over union (IoU) thresholds ranging from 0.5 to 0.95 with a step size of 0.05 (AP_50:95_) is calculated as follows:


(4) $$AP_{50:95}=\frac{1}{10}\left(AP_{50}+AP_{55}+AP_{60}+\cdots +AP_{90}+AP_{95}\right)$$

In this study, the coefficient of determination (R²), root mean squared error (RMSE), mean absolute error (MAE), and mean absolute percentage error (MAPE) were employed as evaluation metrics to comprehensively assess the counting performance of the model. These metrics were selected on the basis of previous studies conducted by Barreto et al. (2021); Chen et al. (2023) and Xue et al. (2024) on the subject of crop counting performance. The R² value represents the proportion of the variance in the observed data that can be explained by the model. A value of R² closer to 1 indicates a superior fit and counting performance of the model. The RMSE is a measure of the magnitude of the discrepancies between the model’s predictions and the actual observations, with greater weight given to larger errors. A lower RMSE value indicates superior model performance. The MAE reflects the mean of the absolute differences between the true and predicted counts. As a non-negative value, a smaller MAE suggests enhanced model performance. The MAPE is a normalized version of MAE, facilitating comparisons across different scales. Lower values of RMSE, MAE, and MAPE indicate enhanced model performance in terms of counting accuracy.


(5) $$R^{2}=1-\frac{\sum_{1}^{n}{\left(m_{i}-p_{i}\right)}^{2}}{\sum_{1}^{n}{\left(m_{i}-{\bar{m}}_{i}\right)}^{2}}$$

(6) $$RMSE=\sqrt{\frac{{\sum_{1}^{n}\left(m_{i}-p_{i}\right)}^{2}}{n}}$$

(7) $$MAE=\frac{1}{n}\sum_{1}^{n}\left|m_{i}-p_{i}\right|$$

(8) $$MAPE=\frac{1}{n}\sum_{1}^{n}\left|\frac{m_{i}-p_{i}}{m_{i}}\right|\times 100\%$$

In the equations above, *m_i_* represents the actual count of early maize seedlings in the images, ${\bar{m}}_{i}$ represents the average actual count of early maize seedlings in the images, *p_i_* represents the count of early maize seedlings predicted by the model, and *n* represents the number of the images.

## 3 Results

### 3.1 Ablation experiment

In order to evaluate the effectiveness of RC-Dino, we conducted ablation studies in this section, in which we tested various modifications to the Dino model on the EMSD dataset. The results of these experiments are presented in **Table 2**, where the presence of checkmarks (✓) indicates the activation of a convolutional layer or module.

**Table 2:** Model ablation experiment.

Modification 1 Transfer learning	Modification 2 RSCconv	Modification 3 ASCFF	Epoch	Recall	AP_50:95_	AP_S_	AP_M_
–	–	–	12	0.720	0.614	0.654	0.714
√	–	–	12	0.759	0.670	0.711	0.770
√	√	–	12	0.770	0.699	0.735	0.809
√	√	√	12	0.779	0.714	0.752	0.824

As evidenced by the results presented in **Table 2**, the proposed modifications to the Dino model have led to the following improvements: The introduction of pre-trained weights for the backbone accelerates the convergence of the model, increasing This resulted in an increase in AP_50:95_ by 3.9% and Recall by 5.6%. Furthermore, the replacement of 3x3 convolutions with RSCconv in the backbone enhances and refines the features of early maize seedlings, resulting in an increase in AP_50:95_ by 1.1% and recall by 2.9%. Finally, replacing ChannelMapper with ASCFF in the Neck integrated features at different scales of early maize seedlings, resulting in an increase in AP_50:95_ by 1.5% and Recall by 0.9%.

### 3.2 Model evaluation for detection

In order to comprehensively evaluate the performance of our improved network model in terms of detection and technical aspects, we conducted comparisons with several state-of-the-art network models. These included two-stage algorithms such as Faster R-CNN, single-stage algorithms like YOLOX and RetinaNet, as well as end-to-end algorithms such as Deformable DETR (Zhu et al., 2020). The parameter settings for these models were maintained in accordance with their original source code. The results of these experiments are presented in **Table 3**.

**Table 3:** Comparison of target detection algorithms.

Model	Epoch	Recall	AP_50:95_	AP_S_	AP_M_
YOLOX	300	0.663	0.592	0.615	0.674
RetinaNet	12	0.474	0.378	0.374	0.410
RetinaNet + RSCconv+ ASCFF	12	0.496	0.408	0.399	0.476
Faster R-CNN	12	0.666	0.605	0.592	0.687
Faster R-CNN + RSCconv+ ASCFF	12	0.713	0.668	0.650	0.790
Deformable DETR	50	0.707	0.640	0.626	0.753
Deformable DETR + RSCconv+ ASCFF	50	0.724	0.659	0.643	0.774
Dino	12	0.720	0.614	0.654	0.714
RC-Dino	12	0.779	0.714	0.752	0.824

The Faster R-CNN, RetinaNet, Deformable DETR, and Dino models all utilized ResNet50 as the underlying foundation. In the case of both Faster R-CNN and RetinaNet, feature maps were extracted from the backbone at Levels 1 to 4. Subsequently, a Feature Pyramid Network (FPN) was employed to perform feature extraction and spatial scaling on the aforementioned feature maps, which were then subjected to classification and localization tasks by means of different detection heads. In contrast, Deformable DETR and Dino extracted feature maps from Level 2 to Level 4. Subsequently, a ChannelMapper was employed for the purpose of fusing the aforementioned feature maps and adjusting the channel dimensions. Subsequently, a Transformer-based detection head was employed for both classification and localization tasks. In contrast, YOLOX employed CSPDarknet as its backbone, extracting feature maps from Level-3, Level-4, and Level-5. The Path Aggregation Feature Pyramid Network (PAFPN) conducted up sampling and down sampling operations on these feature maps to obtain three distinct scales of feature maps. However, this fusion method is more susceptible to the omission of features associated with early maize seedlings.

In comparison to the Deformable DETR and Dino models, the Faster R-CNN and RetinaNet models offer the additional benefit of shallow feature maps in the neck component. However, due to the absence of calibration and optimization of the early maize seedlings features in the backbone, in addition to the failure to integrate multi-scale features in the neck, the deep feature maps generated by the neck contain a sparser representation of the early maize seedlings features than the shallow feature maps. This has an impact on the detection capabilities of these models. The Deformable DETR and Dino models demonstrate superior recall performance compared to the Faster R-CNN and RetinaNet models, which can be attributed to their utilization of multi-head self-attention and deformable attention mechanisms during the detection process.

As demonstrated in **Table 3**, the outcomes of these experiments indicate that RC-Dino exhibits superior performance compared to the other evaluated detection algorithms in terms of recall, average precision (AP), and its constituent subcategories. In comparison to the Dino model, RC-Dino exhibits a notable enhancement, with an 16.29% improvement in AP_50:95_ and an 8.19% increase in recall. Moreover, the integration of RSCconv and ASCFF with the object detection model demonstrates that RSCconv and ASCFF effectively enhance the performance of the Faster R-CNN, RetinaNet and Deformable DETR models in recall, AP_50:95_ and the subcategories of AP.

In light of the aforementioned experiments, it can be posited that the incorporation of RSCconv into the backbone facilitates the amplification, calibration, and optimization of early maize seedlings features within the feature maps. This results in an enhanced representation of small object features in the output of the backbone. Furthermore, the incorporation of ASCFF in the neck enables the adaptive spatial-channel fusion of multi-scale features. This fusion process enhances the representation of early maize seedlings features in deeper feature maps while preserving information in shallower feature maps. Consequently, the subsequent classification and localization detection heads exhibit higher precision in identifying and localizing small objects.

### 3.3 Model evaluation in counting

The aforementioned models have previously been employed in ablation studies and for evaluating target detection. They were also utilized to assess the number of early maize seedlings in the EMSD test dataset. In this context, early maize seedlings with a confidence score exceeding 0.5 were deemed to have been successfully identified. The resulting counts were then compared against the actual number of early maize seedlings. The counting results are presented in **Tables 4** and **5**.

**Table 4:** Counting performance of different models in test dataset A.

Model	Epoch	Ground Truth	Inference Value	R^2^	RMSE	MAE	MAPE
YOLOX	300	2773	3169	0.0537	6.2218	5.2368	14.513
RetinaNet	12	2773	2810	0.5944	4.0733	2.9605	8.0561
RetinaNet + RSCconv+ ASCFF	12	2773	2860	0.7803	2.9978	2.2763	6.2506
Faster R-CNN	12	2773	2926	0.7572	3.1519	2.3553	6.6846
Faster R-CNN + RSCconv+ ASCFF	12	2773	2922	0.7597	3.1351	2.2389	6.5986
Deformable DETR	50	2773	2547	0.5825	4.1327	3.2632	8.6407
Deformable DETR + RSCconv+ ASCFF	50	2773	2570	0.6381	3.8474	3.2500	8.8912
Dino	12	2773	2059	-1.5526	10.2187	9.3947	25.3888
RC-Dino (Dino + Transfer-learning + RSCconv +ASCFF)	12	2773	2334	0.7909	2.9245	2.3684	6.5510

**Table 5:** Counting performance of different models in test dataset B.

Model	Epoch	Ground Truth	Inference Value	R^2^	RMSE	MAE	MAPE
YOLOX	300	8315	8844	0.5811	12.7616	10.3684	7.0831
RetinaNet	12	8315	8371	0.7752	9.3490	7.4386	5.1938
RetinaNet + RSCconv+ ASCFF	12	8315	8264	0.8292	8.1488	6.6491	4.7160
Faster R-CNN	12	8315	8703	0.7181	10.4697	8.4912	5.9029
Faster R-CNN + RSCconv+ ASCFF	12	8315	8600	0.8083	8.6318	7.1053	4.8918
Deformable DETR	50	8315	7919	0.7812	9.2224	7.8246	5.4921
Deformable DETR + RSCconv+ ASCFF	50	8315	8011	0.8295	8.1413	6.807	4.805
Dino	12	8315	6123	-2.9712	39.2924	38.4561	26.2798
RC-Dino (Dino + Transfer-learning + RSCconv +ASCFF)	12	8315	7934	0.8305	8.1186	6.7193	4.6576

A total of 2,773 early maize seedlings were annotated in Test Dataset A. The RC-Dino model demonstrated a high degree of accuracy in counting the 2,368 early maize seedlings, with an R² value of 0.7909, an RMSE of 2.9245, an MAE of 2.3684, and a MAPE of 6.5510. In comparison to the Dino model, the RC-Dino model demonstrated a notable improvement in its R² value, shifting from a negative correlation of -1.5526 to a positive correlation of 0.7909. This signifies a substantial enhancement in the linear relationship between the model’s predictions and the actual values. Additionally, the RMSE exhibited a 78.57% reduction, the MAE an 80.63% reduction, and the MAPE a 77.73% reduction.

A total of 8,315 early maize seedlings were annotated in Test Dataset B. The RC-Dino model was able to successfully count 7,934 early maize seedlings, achieving an R² value of 0.8305, an RMSE of 8.1186, an MAE of 6.7193, and a MAPE of 4.6576. In comparison to the Dino model, the RC-Dino model exhibited a notable enhancement in predictive accuracy, as evidenced by an improvement in the R² value from -2.9712 to 0.8305. This resulted in a transformation from negative to positive correlation. Furthermore, the RMSE decreased by 78.57%, the MAE by 80.63%, and the MAPE by 77.73%.

**Figure 7:**
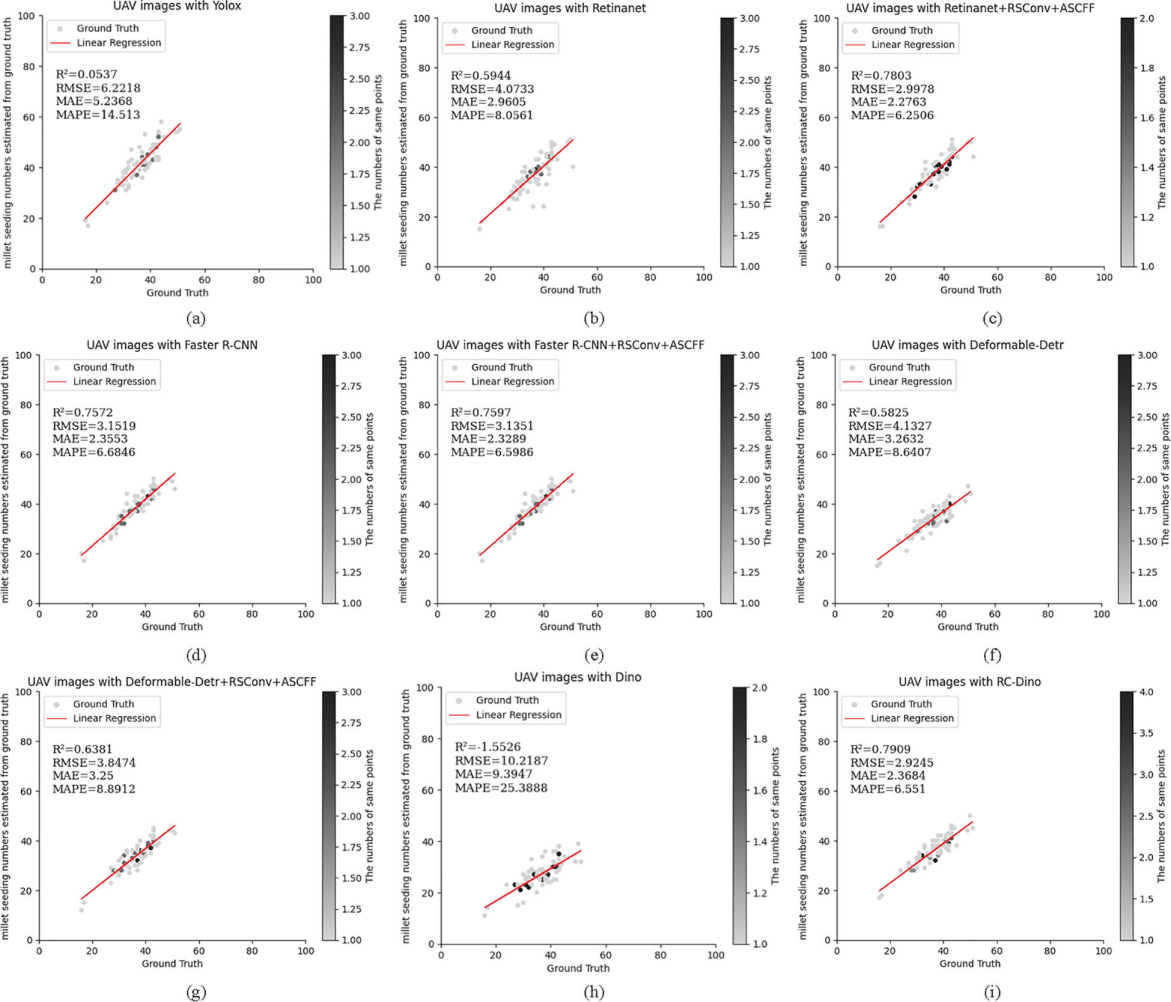
Count statistics for the baseline of the different models and their improved versions for the Test Dataset A: **(A)** UAV images with Yolox (R²=0.0537); **(B)** UAV images with Retinanet (R^2^=0.5944); **(C)** UAV images with Retinanet+RSConv+ASCFF (R^2^=0.7803); **(D)** UAV images with Faster R-CNN (R^2^=0.7572); **(E)** UAV images with Faster R-CNN+RSConv+ASCFF (R^2^=0.7597); **(F)** UAV images with Deformable-Detr (R^2^=0.5825); **(G)** UAV images with Deformable-Detr+RSConv+ASCFF (R^2^=0.6381); **(H)** UAV images with Dino (R^2^=-1.5526); and **(I)** UAV images with RC-Dino (R^2^=0.7909). Each subplot presents ground truth versus predicted counts with corresponding statistical metrics (R^2^, RMSE, MAE, and MAPE) for model evaluation on counting range 0-100.

**Figure 8:**
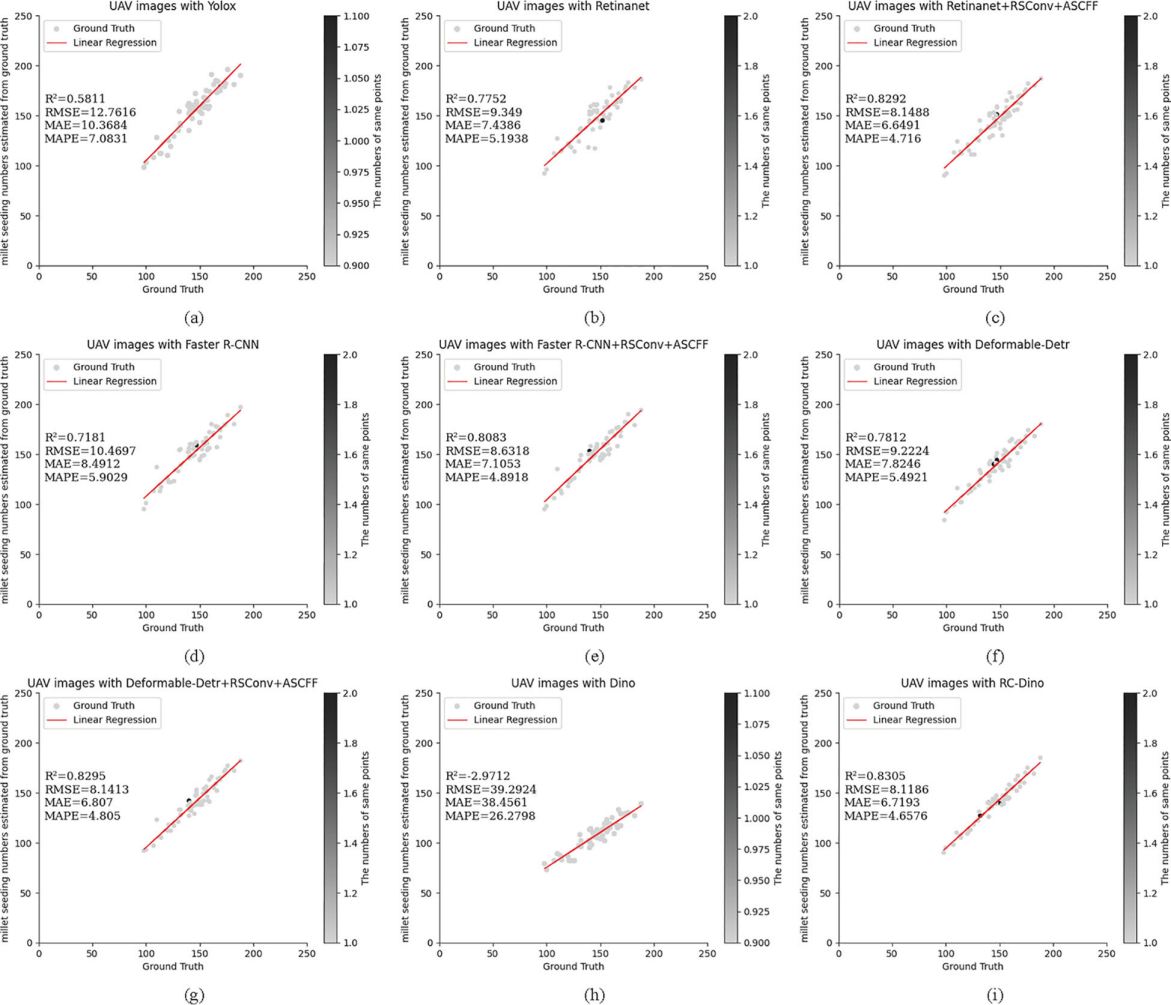
Count statistics for the baseline of the different models and their improved versions for the Test Dataset B: **(A)** UAV images with Yolox (R^2^=0.5811); **(B)** UAV images with Retinanet (R^2^=0.7752); **(C)** UAV images with Retinanet+RSConv+ASCFF (R^2^=0.8292); **(D)** UAV images with Faster R-CNN (R^2^=0.7181); **(E)** UAV images with Faster R-CNN+RSConv+ASCFF (R^2^=0.8083); **(F)** UAV images with Deformable-Detr (R^2^=0.7812); **(G)** UAV images with Deformable-Detr+RSConv+ASCFF (R^2^=0.8295); **(H)** UAV images with Dino (R^2^=-2.9712); and **(I)** UAV images with RC-Dino (R^2^=0.8305). Each subplot presents ground truth versus predicted counts with corresponding statistical metrics (R^2^, RMSE, MAE, and MAPE) for model evaluation on counting range 0-250.

The results of the counting assessment indicate that RC-Dino exhibited the highest accuracy in counting early maize seedlings. **Figures 7** and **8** illustrate the statistical distributions of the counting results for different models. The black dots represent the number of overlapping grey dots, with darker points indicating a greater degree of overlap among the grey dots.

## 4 Analysis

This paper introduces RC-Dino, an early maize seedlings counting method based on adaptive spatial-channel feature fusion and self-calibrated convolution. An Early Maize Seedlings Dataset (EMSD) was created using images captured by drones at an altitude of 12 m, 20 m and 24 m thereby ensuring a large annotation scale and high annotation accuracy. The effectiveness and plug-and-play capability of the proposed RSCconv and the ASCFF module were validated by applying them to other models, thereby enhancing the generalizability of our approach.

### 4.1 Model performance for detection and counting tasks

Combining the results in **Table 4**, it can be seen that the application of RSCconv and ASCFF to the object detection model such as Faster R-CNN, RetinaNet and Deformable DETR, improves the models’ performance metrics, R², RMSE, MAE and MAPE, on the Test Dataset A and Test Dataset B.

### 4.2 Feature loss of detection model

**Figure 9:**
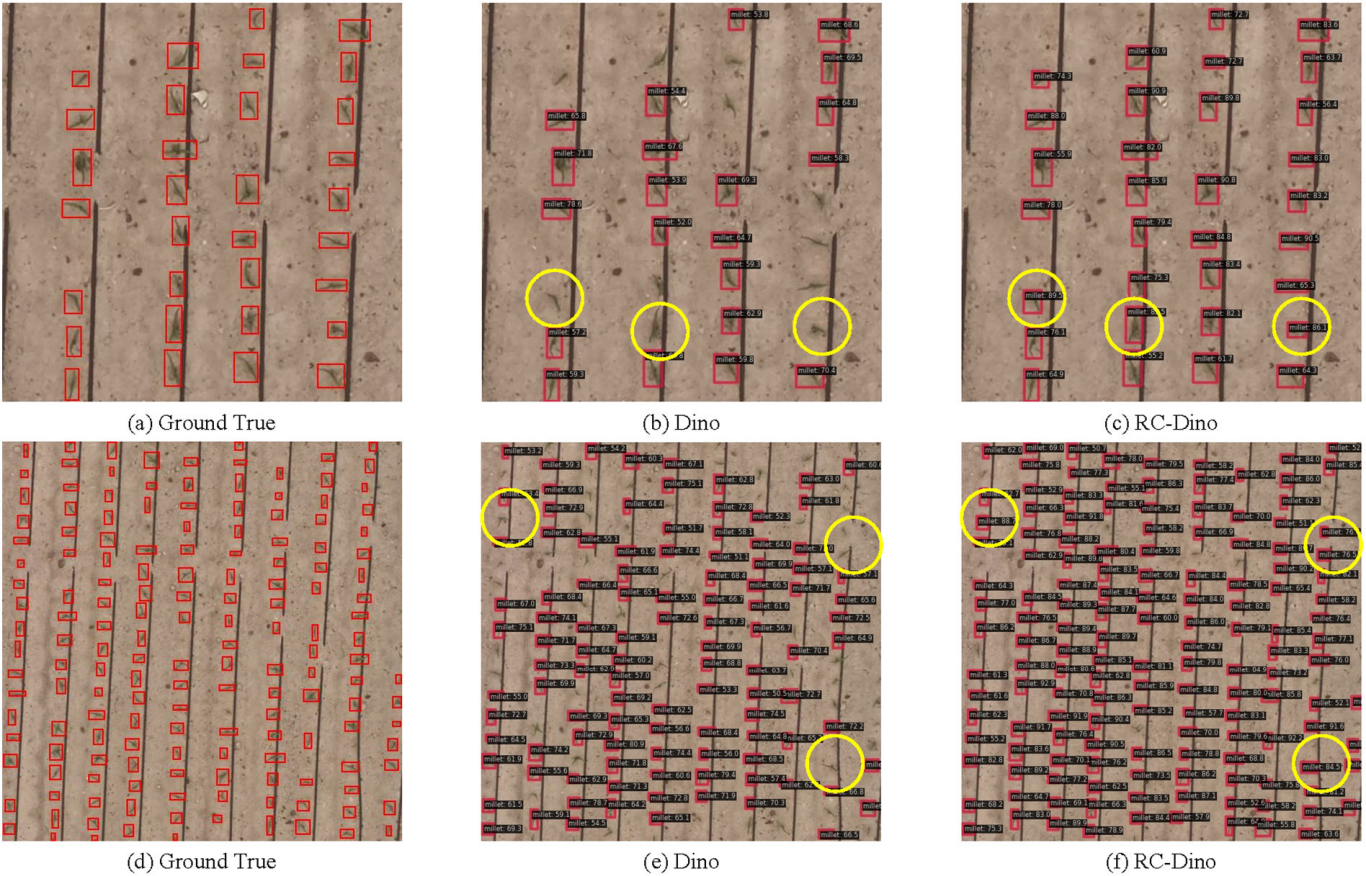
Comparison of detection between Dino and RC-Dino. [Annotated Data are **(A, D)**, Inference Results of Dino are **(B, E)**, Inference Results of RC-Dino are **(C, F)**].

**Figure 9** depicts the counting performance of the RC-Dino model. As illustrated in **Figures 9B, C, E,** and **F**, the RC-Dino model exhibits enhanced detection capabilities for smaller objects in comparison to the Dino model. This improvement in detection enables more accurate counting of early maize seedlings. The incorporation of RSCconv and ASCFF has augmented the model’s capacity to refine, identify, and localize early maize seedlings. Furthermore, the introduction and initialization of pre-trained weights for the backbone have expedited the convergence of the model, thereby enhancing its performance in feature extraction, detection, and the counting of early maize seedlings.

As illustrated in **Figure 9**, our analysis reveals that the majority of counting errors can be attributed to the failure to detect early maize seedlings. The potential reasons for Dino’s failure to detect early maize seedlings can be attributed to the following: the deep feature maps generated by the Backbone are unable to effectively locate the seedlings, which results in the exclusion of seedlings features in the deep feature maps passed to the Neck, in the absence of RSCconv. Consequently, within the Neck, the absence of deep information containing early maize seedlings features negates the presence of such features in the shallow information. This results in either the complete absence of early maize seedlings in subsequent detections or the identification of only those with low confidence due to the absence of early maize seedlings features in the feature maps. After integrating RSConv with the ASCFF module into the Faster R-CNN, RetinaNet and Deformable DETR models (see **Figures A1–A3** in Appendix for specific illustrations), the resulting feature maps not only significantly improve the feature representation of early maize seedlings, but also effectively suppress the feature responses of non-early maize seedlings. This improvement significantly improves the model’s ability to detect actual early maize seedlings and further optimizes the counting performance.

### 4.3 The NMS of detection model

**Figure 10:**
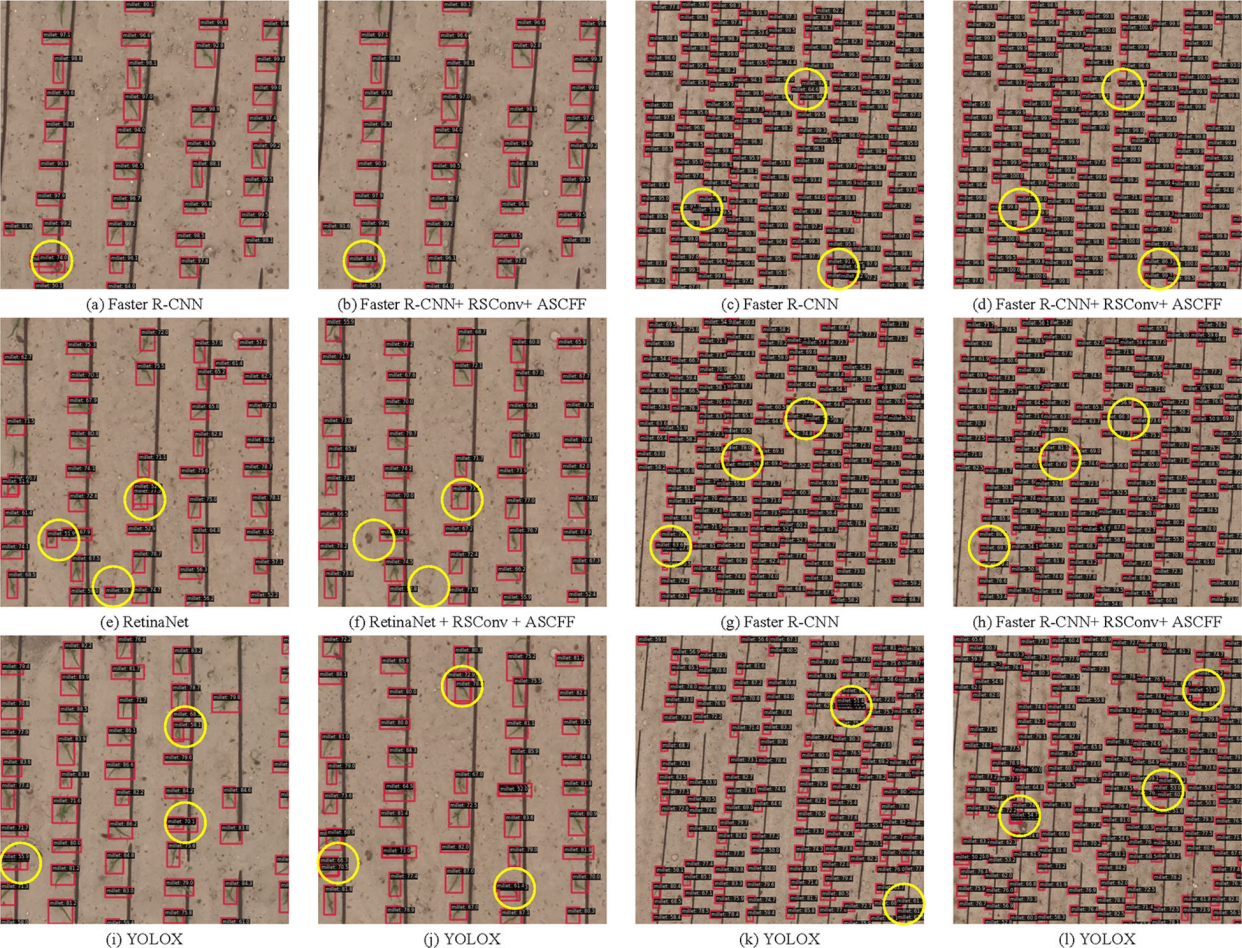
Performance of three models: Faster R-CNN, RetinaNet and YOLOX on the test dataset with default NMS settings.

In the counting results derived from the test dataset, we observed that the Faster R-CNN, YOLOX and RetinaNet models detected a higher number of early maize seedlings compared to the ground truth values (see **Figure 10**). This phenomenon suggests that these models produced an excessive number of bounding boxes, which represent false positives (FPs). A significant part of this problem can be attributed to suboptimal NMS settings. In light of the influence that NMS exerts on counting performance, coupled with the necessity for human expertise and experimental validation when establishing NMS parameters, it is evident that inappropriate NMS settings have the potential to compromise the reliability of counting performance in Faster R-CNN, YOLOX, and RetinaNet.

## 5 Discussion and conclusion

### 5.1 Comparison with existing studies

The RC-Dino model proposed in this study demonstrates superior detection accuracy compared to existing classic models such as Faster R-CNN, RetinaNet, and Deformable DETR. Testing on the Early Maize Seedlings Dataset (EMSD), which comprises 1,233 images and 83,404 annotated seedlings, the results show that the RC-Dino model achieves a 16.29% improvement in AP and an 8.19% improvement in recall. These findings indicate that the RC-Dino model exhibits higher accuracy and robustness in small object detection tasks, particularly in the identification and localization of early maize seedlings, even in complex backgrounds.

The RSCconv and ASCFF modules are designed to be independent of the backbone network, allowing them to be seamlessly integrated into various deep learning models. Experimental results demonstrate that incorporating these modules into models such as Faster R-CNN, RetinaNet, and Deformable DETR leads to significant performance improvements. This suggests that the RSCconv and ASCFF modules possess good generalizability and flexibility, making them suitable for a wide range of tasks and applications. Additionally, the use of pre-trained weights accelerates model convergence, effectively reducing training time and computational resource requirements.

RSCconv enhances the distinction between early maize seedlings and other background elements by adaptively calibrating spatial domain features, thereby reducing noise interference. ASCFF, on the other hand, retains more local detail information through multi-scale feature fusion, further enhancing the model’s feature representation capabilities. These improvements ensure that the model maintains high detection accuracy across images captured at different drone flight altitudes, effectively addressing the issue of unstable image resolution caused by variations in flight height.

In terms of the dataset, we have created the Early Maize Seedlings Dataset (EMSD) using images captured by drones at a height of 12 m, 20 m and 24 m, ensuring a large scale of annotation and high annotation accuracy. The EMSD contains accurate manual annotations for a total of 83,404 early maize seedlings. In contrast, previous studies (Jin et al., 2017; Ghosal et al., 2019; Kitano et al., 2019; Pang, 2020; Vong et al., 2021; Barreto et al., 2021; Chen et al., 2023) reported limited counting accuracy, possibly due to lower drone flight heights (mostly between 3-10 m, with two papers using 20 m and one using 30 m) and smaller dataset sizes.

### 5.2 Study limitations

First, the EMSD used in this study was derived from a single data source, which may limit the generalizability of the model. Furthermore, since the EMSD mainly consists of annotated information for early maize seedlings under specific varieties and growing conditions, the generalizability of the model to different varieties and different environmental conditions requires further validation. Future research could consider training and validating the model with multi-source data to improve its generalizability.

Secondly, the robustness of the model to factors such as lighting variation and occlusion needs to be improved. Lighting variations are a common challenge in practical applications and can affect the counting accuracy of the model. To improve the robustness of the model, future work could introduce more advanced light invariant algorithms.

Third, for early maize seedlings with significant morphological variation, the proposed method may not accurately identify and count them. Morphological variation can result from differences in growth processes, pest or disease effects, or other factors. To improve counting accuracy in such scenarios, future research could consider incorporating more morphology-related features or using morphology-adaptive algorithms to refine counting accuracy.

Finally, although the method is primarily focused on the task of counting early maize seedlings, there is room for further exploration in terms of classification of early maize seedlings and analysis of other growth parameters. Future research could integrate it with tasks such as maize growth monitoring and yield prediction to broaden the scope of the method and provide more comprehensive technological support for agricultural production.

### 5.3 Conclusion

This paper presents a deep learning-based method, the RC-Dino model, for accurate detection and counting of early maize seedlings under field conditions. The model improves the accuracy of small object detection by incorporating RSCconv in the backbone network, using pre-trained backbone weights, and introducing ASCFF in the neck. The RC-Dino model excels in early maize seedlings detection, achieving higher accuracy within the same training cycle. This method has significant potential applications in early maize seedlings counting using UAV, planting density estimation, yield prediction and intelligent field management. In particular, the RC-Dino model is not limited to the detection and counting of early maize seedlings from UAV images, but can be extended to other common crops with similar characteristics. Moreover, the RSCconv convolution and ASCFF modules in RC-Dino not only improve the accuracy of the Dino model’s detection, but also have the potential to improve the detection and enumeration capabilities of a variety of other object detection models.

## Data Availability

The datasets presented in this article are not readily available due to the fact that the dataset was manually annotated by many people over a significant period of time, and because we need to continue expanding and improving this dataset for further research, we are unable to make it publicly available at this time. However, our code is open to the public. The RSCconv and ASCFF code mentioned in this paper can be found here: https://github.com/collapser-AI/RC-Dino. Requests to access the datasets should be directed to https://github.com/collapser-AI/RC-Dino.
