# Supplementary figures and images for: Adaptive spatial-channel feature fusion and self-calibrated convolution for early maize seedlings counting in UAV images
Source: Front Plant Sci. 2025 Feb 3;15:1496801. doi: 10.3389/fpls.2024.1496801 (PMC11841422; doi:10.3389/fpls.2024.1496801)

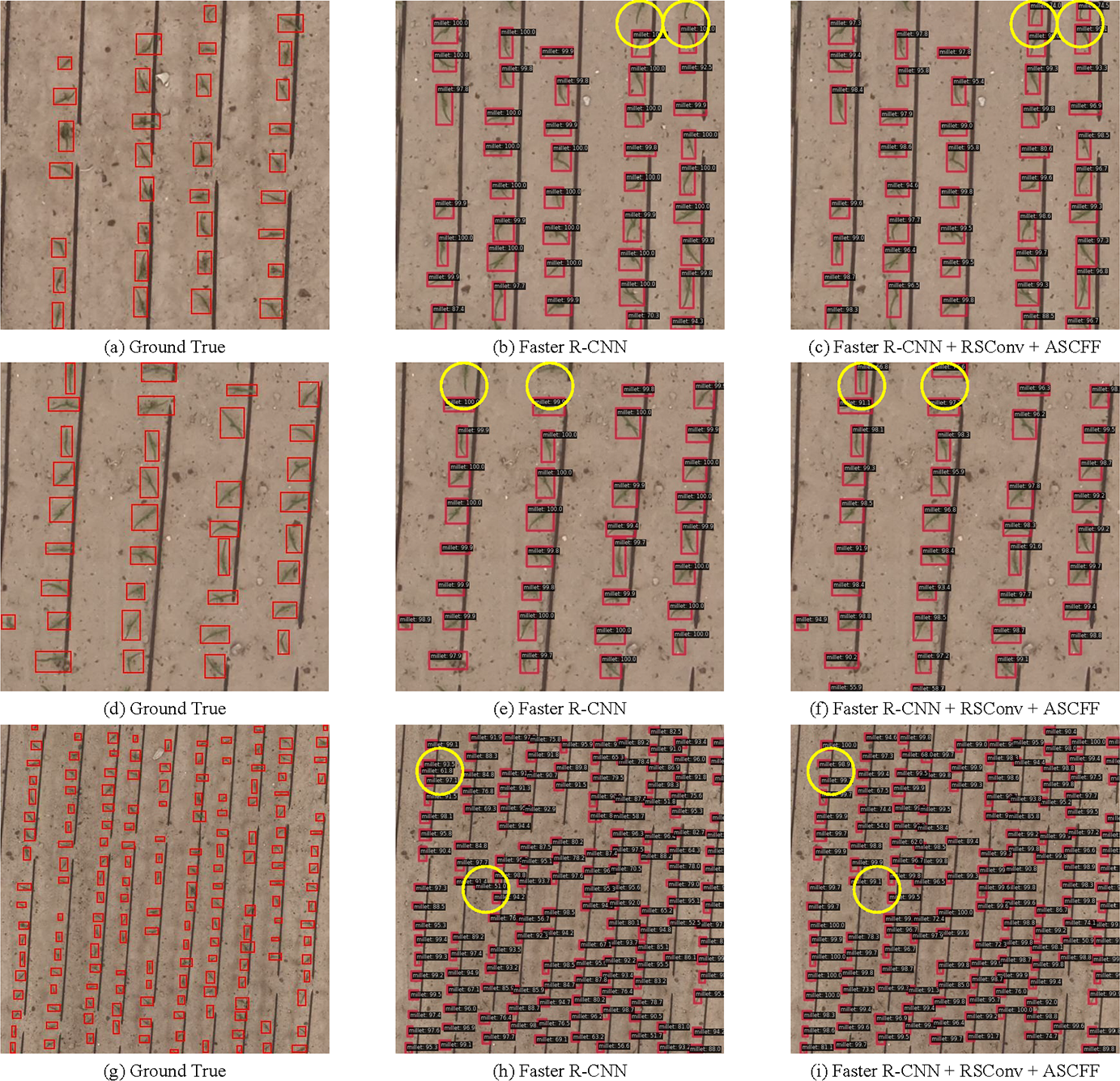

Supplement: Supplementary file 1 [file Image1.tif]

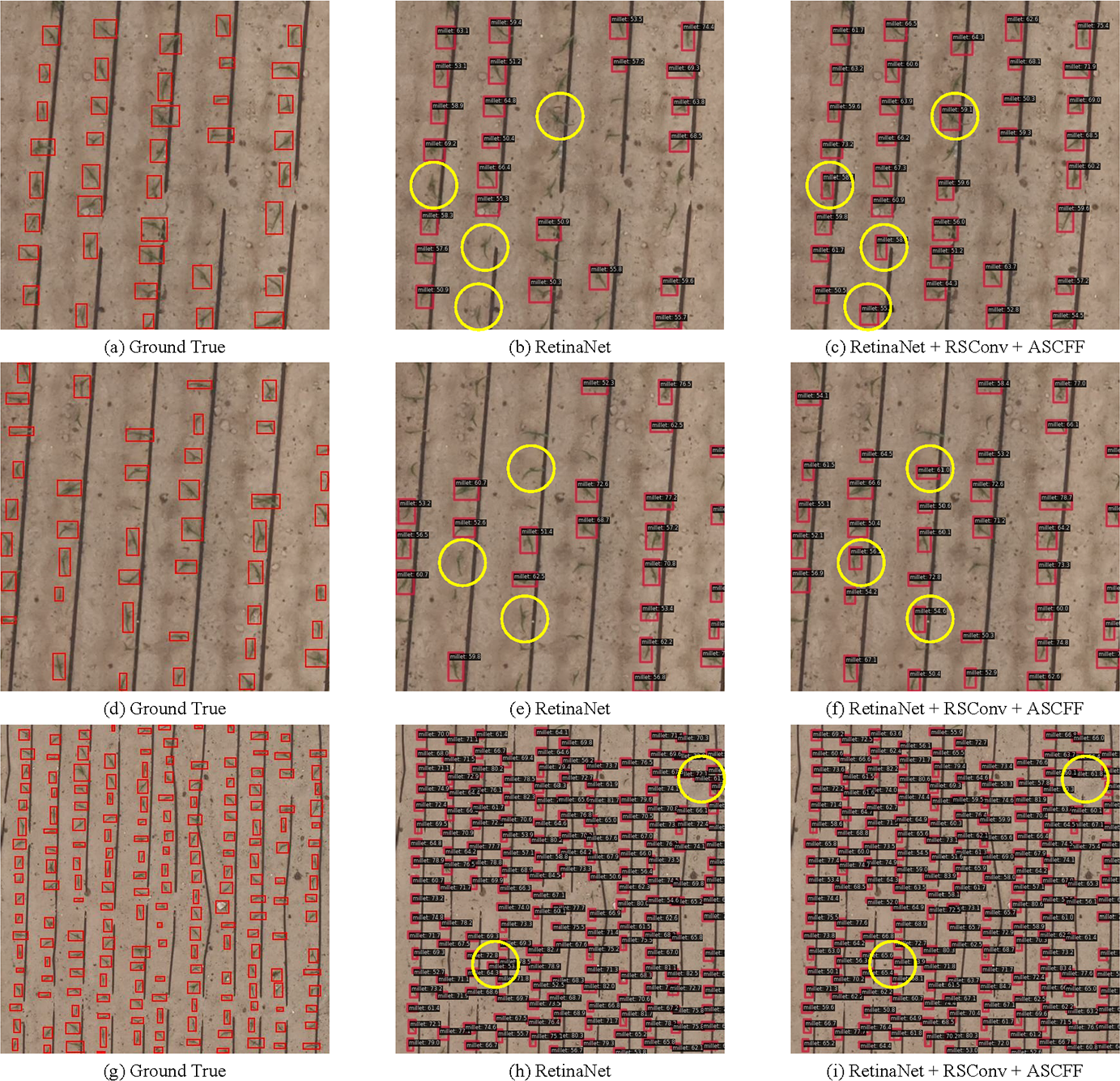

Supplement: Supplementary file 2 [file Image2.tiff]

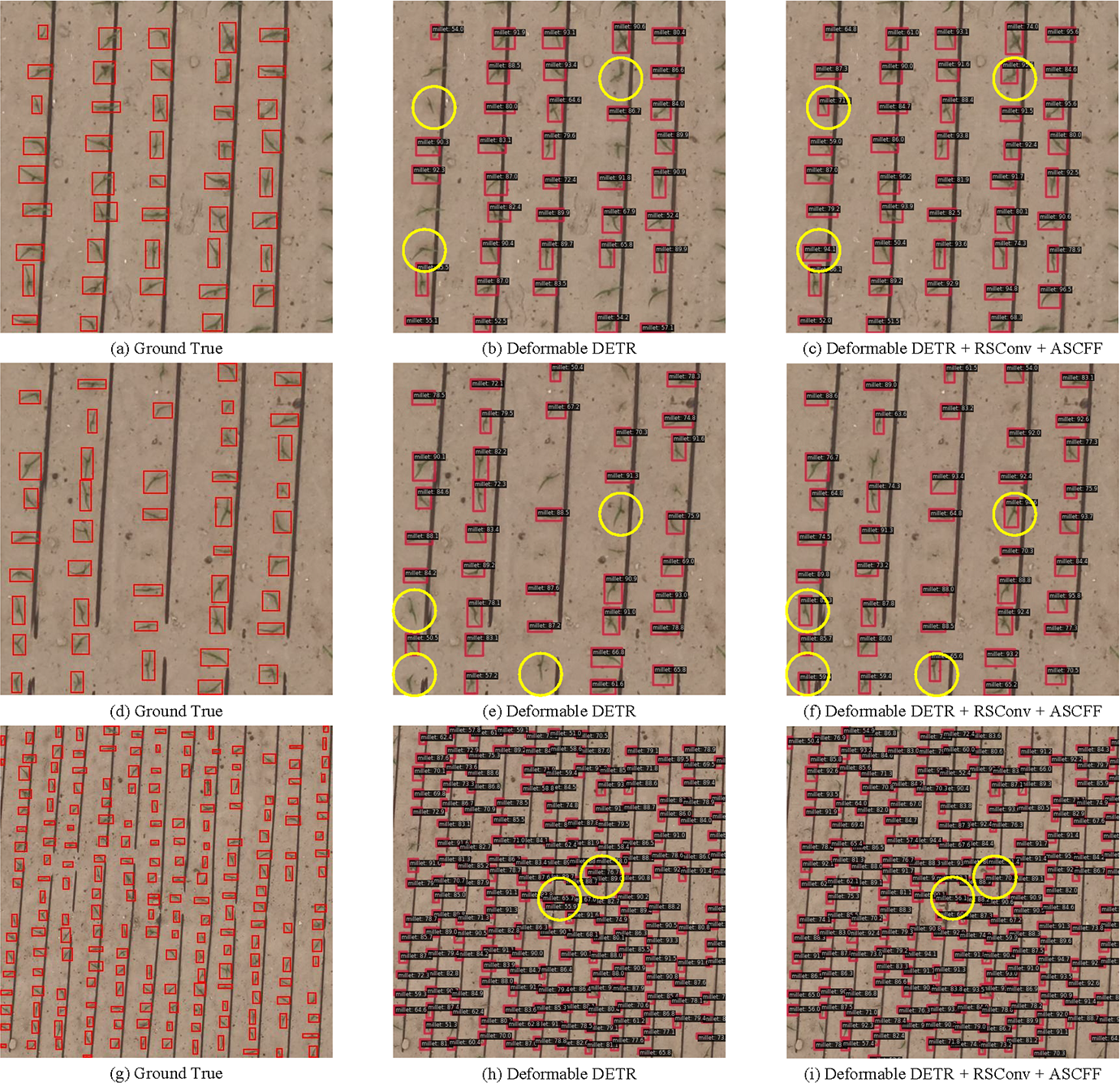

Supplement: Supplementary file 3 [file Image3.tiff]
